# Essential Roles of Natural Products and Gaseous Mediators on Neuronal Cell Death or Survival

**DOI:** 10.3390/ijms17101652

**Published:** 2016-09-29

**Authors:** Yoshinori Mikami, Sho Kakizawa, Toshiko Yamazawa

**Affiliations:** 1 Department of Physiology, School of Medicine, Faculty of Medicine, Toho University, 5-21-16 Omori-Nishi, Ota-ku, Tokyo 143-8540, Japan; ymikami@med.toho-u.ac.jp; 2 Department of Biological Chemistry, Graduate School of Pharmaceutical Sciences, Kyoto University, 46-29 Yoshida-Shimoadachi-cho, Sakyo-ku, Kyoto 606-8501, Japan; kakizawa.sho.4u@kyoto-u.ac.jp; 3 Department of Molecular Physiology, The Jikei University School of Medicine, 3-25-8 Nishishimbashi, Minato-ku, Tokyo 105-8461, Japan

**Keywords:** chlorogenic acid, glutamate neurotoxicity, hydrogen sulfide, neuroprotection, nitric oxide

## Abstract

Although precise cellular and molecular mechanisms underlying neurodegeneration still remain enigmatic, key factors associated with degenerative disorders, such as glutamate toxicity and oxidative stress, have been recently identified. Accordingly, there has been growing interest in examining the effects of exogenous and endogenous molecules on neuroprotection and neurodegeneration. In this paper, we review recent studies on neuroprotective and/or neurodegenerative effects of natural products, such as caffeic acid and chlorogenic acid, and gaseous mediators, including hydrogen sulfide and nitric oxide. Furthermore, possible molecular mechanisms of these molecules in relation to glutamate signals are discussed. Insight into the pathophysiological role of these molecules will make progress in our understanding of molecular mechanisms underlying neurodegenerative diseases, and is expected to lead to potential therapeutic approaches.

## 1. Introduction

There are a wide variety of neurodegenerative diseases, such as Alzheimer’s disease, Parkinson’s disease, and stroke, with distinct symptoms and pathologies. For many of these diseases, the vast majority of cases are sporadic. Therefore, the need for research on pathogenesis, therapeutic agents, and useful biomarkers are increasing. Oxidative stress is recognized as one of the critical factors in many neurodegenerative diseases under conditions of hypoxia/ischemia [1,2,3]. Antioxidant compounds derived from natural products have demonstrated neuroprotective activity in models of neuronal cell death and neurodegeneration in vitro and in vivo [4].

Glutamate is the most common neurotransmitter in the central nervous system, and plays an important role in signal transduction and functional regulation, including synaptic plasticity. On the other hand, it is widely believed that glutamate may also be responsible for quite a variety of diseases of the central nervous system. Brain injury and stroke lead to an excess of glutamate, and excessive glutamate is actually toxic to neurons, and multiple mechanisms underlying glutamate-induced neurotoxicity [5,6]. Accordingly, glutamate excitotoxicity, oxidative stress, and mitochondrial dysfunction are common features that lead to neuronal cell death in cerebral ischemia, Parkinson’s disease, and Alzheimer’s disease [2,5,6]. In ischemic stroke, neuronal excitotoxicity, caused by increased extracellular glutamate levels, is known to result in Ca^2+^ overload [7]. *N*-methyl-d-aspartate (NMDA) receptor, a type of the ionotropic glutamate receptor, is linked to downstream neurotoxic proteins, such as neuronal nitric oxide synthase (nNOS), through the postsynaptic density 95 (PSD95), discs large, zonula occludens-1 (PDZ) domains of PSD95 [8]. The activation of nNOS leads to the production of nitric oxide (NO) and reactive oxygen species (ROS) which, in turn, leads to neuronal cell death [8,9,10,11,12,13]. In addition to its role in neuronal toxicity, NO, which is produced in many tissues [14], is a signaling molecule and activates cyclic guanosine monophosphate (GMP)-dependent protein kinase pathway [15]. In addition, NO regulates the function of various target proteins through *S*-nitrosylation, a covalent reaction with a cysteine thiol group on the target protein [16,17,18].

**Figure 1:**
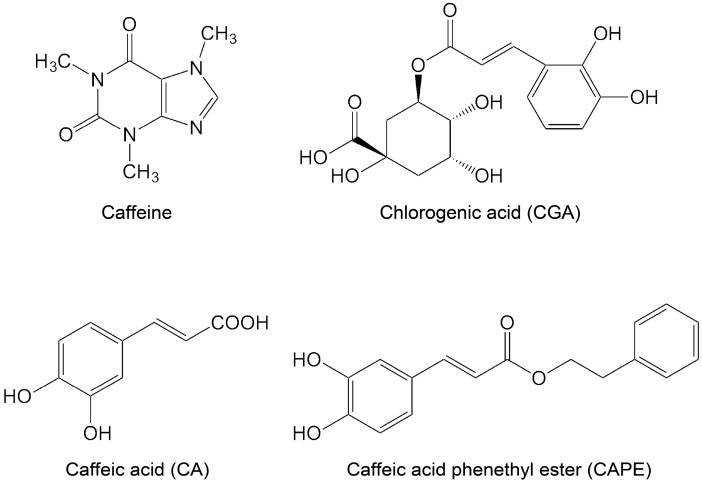
Structures of coffee components.

There are many natural products and endogenous factors that protect neurons against cell death. We are dependent on natural products to maintain redox homeostasis, at least in part. Ascorbate, which is also known as vitamin C, is a radical scavenging antioxidant [19,20]. In most mammals, ascorbate precursor 2-keto-l-glono-1,4-lactone is produced by l-glono-1,4-lactone oxidase from l-gulono-1,4-lactone, which is derived from uridine-5′-diphosphoglucose (UDP-glucose). Since humans and other primates have lost the ability to produce ascorbate, they are dependent on its dietary intake. In addition to vitamin C, other natural products, such as vitamin E (tocopherols and tocotrienols), vitamin A, and carotenoids, function as antioxidants [21]. Carotenoids, which are produced in red or yellow fruits and vegetables, are the most abundant plant-derived compounds. Natural polyphenols are the largest group of phytochemicals, and have antioxidant, cardioprotective, anticancer, anti-aging, and anti-inflammatory properties [22,23]. They are found in many plants, such as grapes, olives, and blueberries, and in plant-based foods, such as coffee, green tea, and wine [22]. In the former part of this paper, we review the natural polyphenols caffeic acid (CA) and chlorogenic acid (CGA) (Figure 1), and their roles in protecting neurons from excitotoxicity. CA is a known antioxidant that is present in coffee, wine, and green tea. CGA is also a well-known antioxidant that is present in green tea and roasted coffee. CGA protects neurons against oxidative stress and glutamate-induced neuronal cell death.

Glutathione (γ-glutamylcysteinylglycine) is a tripeptide comprised of glutamic acid, cysteine, and glycine, and the most abundant endogenous cytoprotectant factor [1,24,25]. GSH, the reduced form of glutathione, is a major endogenous antioxidant molecule with concentrations reaching millimolar levels (1–10 mM) and micromolar levels (10–30 µM) levels, in cells and plasma, respectively [26,27]. GSH is considered to be one of the most important scavengers of ROS. In addition to GSH, many other small, water-soluble antioxidants can also act as ROS scavengers in the cell. α-Lipoic acid is a sulfur-containing antioxidant that is synthesized in the mitochondrion by lipoic acid synthase, and can be absorbed from food or supplements [28,29]. Both the oxidized form (α-lipoic acid) and reduced forms (dihydrolipoic acid; DHLA) can play a role as an antioxidant [30]. In addition to the natural products and small sulfur-containing molecules described above, some gaseous messengers are reported to exert neuroprotective effects. Especially, hydrogen sulfide (H_2_S) is well known to play a role in cytoprotection, in addition to its role as a signaling molecule, for instance, in facilitating the induction of hippocampal long-term potentiation (LTP) [31]. Therefore, in this review, we also outline endogenous gaseous mediators that regulate neuronal cell death or survival.

## 2. Neuroprotection by Coffee Polyphenols

### 2.1. Coffee Consumption and Health

Coffee is one of the most popular beverages and consists of a complex mixture of chemicals, including caffeine, CGA, and CA [32] (Figure 1). Habitual coffee consumption has many effects on cardiovascular health, such as the reduction of the risk of stroke and anti-inflammatory diseases [33,34,35].

Epidemiological studies show that habitual coffee consumption reduces the risk of ischemic stroke [36]. A large, prospective cohort study indicates the dose-dependent inverse association between coffee consumption and death due to heart disease, respiratory disease, and stroke [34]. A meta-analysis revealed that a consumption of three to six cups of coffee per day is inversely associated with the risk of cardiovascular disease and stroke [37]. In addition, higher rates of coffee consumption also reduced the risk of cardiovascular disease and strokes [37]. Larsson et al. assessed the association between coffee consumption and the risk of stroke in a Swedish mammography cohort [38]. In the age-adjusted analysis, there was no association between coffee consumption and risk of stroke. However, after adjustment for smoking and other risk factors, women who consumed 1–2 cups, 3–4 cups, or >5 cups of coffee per day had a significantly lower risk of stroke compared with those who drank <1 cup of coffee per day [38]. They also reported that coffee consumption was associated with decreased risk of cerebral infarction [38]. In the nurses’ health study, women, without a history of stroke, coronary heart disease, diabetes, or cancer at baseline, who regularly consumed coffee, had a modestly reduced risk of stroke, in comparison to those who did not consume coffee [39]. Thus, daily coffee consumption seems to be beneficial for the reduction of the risk of stroke.

### 2.2. Caffeic Acid

CA, one of the phenolic acids, is widely distributed in higher plants, such as grape, olives, apples, and coffee beans. CA can act as antioxidants by scavenging free radicals [40]. Alzheimer’s disease is a progressive neurodegenerative disorder that can be characterized pathologically by the accumulation of amyloid plaques in the neurons of Alzheimer’s disease patients. The progressive accumulation of β-amyloid (Aβ) forms these senile plaques. Increased production of Aβ and the aggregation of Aβ have been reported to trigger neurotoxicity [1]. In rat pheochromocytoma PC12 cells, which are used as a model for neurons, CA can protect cells against Aβ-induced toxicity in a dose-dependent manner [41]. CA prevents the increase of intracellular Ca^2+^ concentrations induced by Aβ [41], and decreases the Aβ-induced phosphorylation of tau protein and glycogen synthase kinase-3β [41]. The inhibition of acetylcholinesterase and butyrylcholinesterase activity has been used as a therapeutic strategy against Alzheimer’s disease [42]. In the lysates of rat cerebral tissue, CA shows an inhibitory effect on acetylcholinesterase and butyrylcholinesterase [43]. In addition to CA, caffeic acid phenethyl ester (CAPE) (Figure 1) is also an antioxidant flavonoid found in propolis, which is made by honeybees to build their hives [44,45]. CA and CAPE are known neuroprotectants against neurodegeneration. CAPE protects cerebellar granule neurons against glutamate neurotoxicity through the inhibition of caspase-3 activation and suppression the phosphorylation of p38 [46]. CAPE also protects neurons against the neurotoxicity induced by MPP^+^ (1-methyl-4-phenylpyridinium) by increasing the expression of growth-associated protein 43 (GAP43), synapsin I, and synaptophysin [47]. In HT22 mouse hippocampus cells, CA and CAPE show protective effects against acrolein-induced neurotoxicity [41,48]. Kim et al. reported that CA has a protective effect in an Aβ_25–35_-injected Alzheimer’s disease mouse model: CA showed an improvement of memory deficits and cognitive impairment [48]. They also reported that CA inhibits lipid peroxidation, which occurs due to oxidative stress in the brain, compared with the Aβ_25–35_-injected control group [48]. In an animal model of Parkinson’s disease generated by injection of 6-hydroxydopamine into the rat brain, CAPE prevents dopaminergic neuronal cell loss [49]. Further, Liang et al. reported that CA protects cerebral damage against ischemia-reperfusion injury in rats [50]. These reports demonstrate the neuroprotective role of CA and CAPE, highlighting their potentials for treatment of neurodegenerative diseases.

### 2.3. Chlorogenic Acid

In this section, we focus on the neuroprotective effects of caffeine and CGA. Caffeine, a key component of coffee, is a purine alkaloid. Since the chemical structure of caffeine is similar to adenosine, caffeine is a known antagonist of adenosine receptor and blocks the regulatory effects of adenosine, a potent endogenous neuromodulator, driving the neuroprotective effect of caffeine [51,52]. Epidemiological studies have linked caffeine consumption to a reduced risk of Parkinson’s disease [53,54].

CGA is one of the most abundant polyphenol compounds in coffee, which is one of the major sources of CGA [55,56,57]. Moreover, CGA has a number of beneficial biological activities to reduce the risk of human chronic diseases such as inflammation, cancer, diabetes, and cardiovascular diseases. In addition, CGA has anti-oxidative stress property [58,59,60,61]. In a rat model of transient middle cerebral artery occlusion (MCAO), intraperitoneal administration of CGA reduced infarct volume and sensory-motor functional deficits [62].

**Figure 2:**
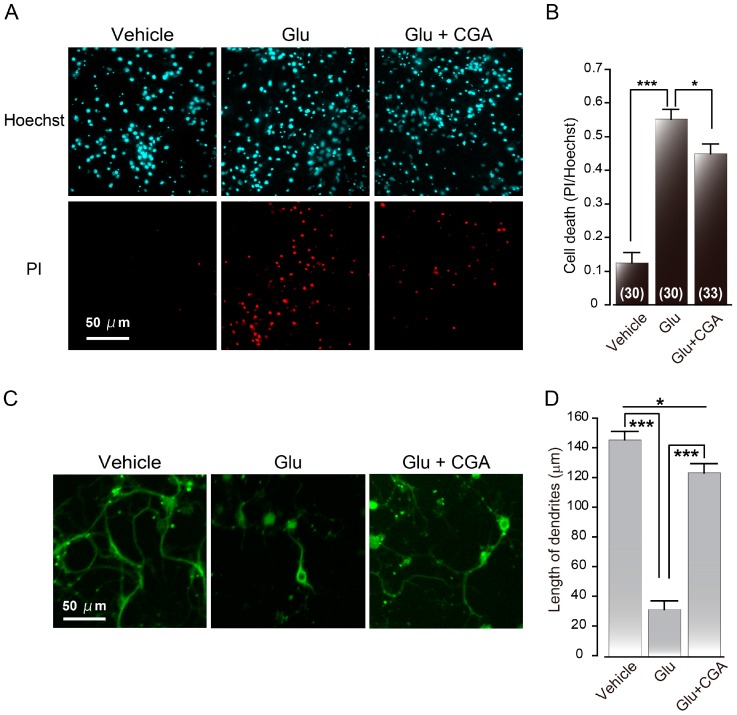
Effects of chlorogenic acid (CGA) on glutamate-induced neuronal cell death. (**A**) The extent of cell death was expressed as a ratio of the number of propidium iodide (PI)-positive cells to that of Hoechst-positive cells; (**B**) numbers in parentheses (30–33) indicate the number of determinations in each condition using different cultures; (**C**) cultured neurons stained with anti-β-III tubulin antibody. Neuronal cell death of cultured cerebral mouse neurons that were assayed following 16 h of treatment with 300 µM glutamate (Glu) without or with 10 µM CGA. Fluorescent immunohistochemistry was based on a modification of a previously described procedure [68,69,70]. All fluorescence microscopy images were obtained by laser confocal microscopy using a TCS SP8 (Leica Microsystems GmbH, Wetzlar, Germany). ImageJ software (National Institute of Health, Bethesda, MD, USA) was used to merge the obtained images; and (**D**) the length of the longest neurites of surviving neuronal cells was analyzed (number of cells: vehicle = 87; Glu = 38; CGA = 42). Data are expressed as mean ± standard error of mean (s.e.m.) * *p* < 0.05; *** *p* < 0.0001, *t*-test compared with treatment of 300 µM Glu. Permission from Elsevier for (**A**,**B**): Mikami, Y.; Yamazawa, T. Chlorogenic acid, a polyphenol in coffee, protects neurons against glutamate neurotoxicity. *Life Sci.* **2015**, *139*, 69–74 [68].

Given its neuroprotective effect in PC12 cells, CGA is one of the candidate components in coffee that may protect neurons from degeneration [59,63,64]. CGA shows a cytoprotective effect against various oxidative stressors, such as tert-butyl hydroperoxide, hydrogen peroxide (H_2_O_2_) and FeSO_4_ [64]. CGA inhibits H_2_O_2_-induced nuclear condensation and DNA fragmentation, hallmarks of apoptotic cell death, in PC12 cells [63]. Cho et al. also reported that H_2_O_2_-induced apoptosis might be prevented by CGA through the poly (ADP-ribose) polymerase (PARP) cleavage and the downregulation of Bcl-X_L_ and caspase-3 expression [63]. In PC12 cells, CGA reduces intracellular accumulation of ROS and prevents H_2_O_2_-induced activation of JNK and p38 MAPK pathways. Further, CGA was also found to have a direct radical scavenger effect on hydroxyl radical, by using electron spin resonance, in combination with spin trapping techniques [61]. These results indicated that CGA works as a ROS scavenger for neuroprotection from oxidative stress. Further, CGA protects against apoptosis that is induced by methylmercury (MeHg) in PC12 cells [59]. MeHg is a highly neurotoxic chemical and induces ROS formation in the brain [59,65,66]. Since MeHg-induced neurotoxicity in neuronal culture is blocked by antioxidants and NMDA receptor antagonists, CGA protects neurons from MeHg by scavenging ROS and/or blocking glutamate receptor. There are also other reports about the neuroprotective effects of CGA in primary cultured neurons from mice or rats. CGA and its metabolite *m*-coumaric acid promotes neuronal differentiation and induce neurite outgrowth in primary cultures derived from rat fetal hippocampus [67]. In our work, primary cultured neurons derived from mouse cerebral cortex, CGA protected neurons from glutamate-induced neuronal cell death [68]. We performed double staining with propidium iodide (PI) and Hoechst 33342 for identification of late neuronal cell death by glutamate. PI is a commonly used marker to detect apoptotic/necrotic cells. The number of PI-positive cells was increased by application of glutamate (Figure 2A,B) [68]. However glutamate-induced neuronal cell death was attenuated in the presence of CGA (Figure 2A,B) [68]. These results indicated that CGA protects neurons from glutamate-induced neuronal cell death. [68]. We also analyzed the morphology of neurons by using anti-β-III tubulin antibody. Glutamate-treated neurons had short, shrunken neurites (Figure 2C,D). The length of dendrites was reduced by treatment with glutamate and this reduction was reversed by CGA administration (Figure 2C,D). CGA decreases the influx of Ca^2+^ through the glutamate receptors [68]. These results suggest that CGA can protect neurons by inhibiting glutamate receptors, which, in turn, inhibits the excessive influx of intracellular Ca^2+^ [68]. Thus, CGA may be a potent therapeutic agent for the prevention of neuronal cell death caused by ischemic stroke.

## 3. Neuroprotection by H_2_S

### 3.1. H_2_S and Polysulfide

H_2_S, which is well known as a toxic gas, is a signaling molecule. It is detected at relatively high concentrations in the brain [71,72,73]. In addition to its role as a signaling molecule, H_2_S is also a known cytoprotectant.

H_2_S is produced from l-cysteine, via the following enzymes: cystathionine β-synthase (CBS), cystathionine γ-lyase (CSE) and 3-mercaptopyruvate sulfurtransferase (3MST) along with cysteine aminotransferase (CAT) [31,74,75]. Thioredoxin (Trx) and dihydrolipoic acid (DHLA) are endogenous reducing cofactors that drive H_2_S release from 3MST [76]. H_2_S is also produced from d-cysteine by 3MST along with d-amino acid oxidase (DAO) [77]. Hydrogen polysulfides (H_2_S_n_; where *n* = 3–7; *n* = 2 is termed as persulfide) are potential H_2_S-derived signaling molecules, which have a higher number of sulfane sulfur atoms than H_2_S [78,79]. In the brain, H_2_S_3_ and H_2_S are produced from 3-mercaptopyruvate (3MP) by 3MST [79].

H_2_S functions as a neuromodulator in the brain. It enhances the activity of NMDA receptors and facilitates the induction of hippocampal LTP, a synaptic model of memory [31]. H_2_S induces Ca^2+^ waves in primary cultured astrocytes by increasing intracellular concentrations of Ca^2+^ [80]. In blood vessel, H_2_S, which is released from endothelial cells, relaxes vascular smooth muscle [74,81,82,83]. There are also some studies indicating the influence of H_2_S on blood pressure in vivo; although, these results remain controversial [82,84].

### 3.2. Neuroprotective Effect of H_2_S

The toxicity of H_2_S was recognized 300 years ago, when the Italian physician Bernardino Ramazzini, who is known as the father of Occupational Medicine, published an account of H_2_S poisoning in 1713. However, interestingly, H_2_S also has cytoprotectant properties.

In primary rat cortical neurons, H_2_S protects neurons against glutamate toxicity [85]. Glutamate can induce cell death by decreasing the total levels of glutathione (GSH and GSSG, the reduced form), a major anti-oxidant. H_2_S can recover glutamate-induced decreases of intracellular glutathione concentrations [85]. H_2_S enhances the activity of γ-glutamylcysteine synthetase to produce γ-glutamylcysteine, a substrate of glutathione synthetase [85]. H_2_S enhances the activity of cystine/cysteine antiporter and increases cysteine transport into the neurons, which also leads to the increase in the levels of γ-glutamylcysteine and glutathione [85,86]. In the murine neuroblastoma Neuro2a cells expressing a mitochondrial H_2_S-producing enzymes 3MST and CAT, showed a significant resistance to oxidative glutamate toxicity, compared with cells transformed with empty vector [86]. H_2_S also protected fetal brain cells by reinstating glutathione levels, which were previously decreased by ischemia-reperfusion in utero [86]. These results indicated that H_2_S protects neurons by increasing the levels of glutathione and directly suppressing ROS in the mitochondria.

In the glial cell line SH-SY5Y, H_2_S scavenges peroxynitrite and oxidant hypochlorous acid (HOCl) and protects cells against peroxynitrite- and HOCl-mediated oxidative damage [87,88].

In addition, H_2_S is known to inhibit apoptosis. Using a model of myocardial ischemia-reperfusion, it was reported that H_2_S limits myocardial infarct size, and preserves left ventricular structure and function in vivo [89]. H_2_S reduces cardiomyocyte apoptosis in vitro and in vivo after myocardial ischemia-reperfusion [89]. It increases the nuclear localization of nuclear factor erythroid-2 related factor 2 (Nrf2), a key regulator of the antioxidant response to protect against oxidative stress [90,91,92]. In cultured mouse neuroblastoma Neuro2A cells, polysulfide exerts a protective effect against *t*-buthylhydroperoxide-induced damage through Nrf2 signaling [93].

In the mouse hippocampal cell line HT22, H_2_S protects neurons from oxidative glutamate toxicity by activating ATP-dependent K^+^ (K_ATP_) channels to stabilize the membrane potential [94]. This cytoprotective mechanism of H_2_S is also seen in the cardiomyocyte ischemic-reperfusion injury model [95]. H_2_S also enhances the cystic fibrosis transmembrane conductance regulator (CFTR) Cl^−^ channel [94]. The activation of K_ATP_ and CFTR Cl^−^ channels by H_2_S is considered to be independent of the increase in the GSH levels.

H_2_S producing ubiquitous enzymes 3MST and CAT are also localized retinal neurons, and the production of H_2_S by these enzymes is regulated in a Ca^2+^-dependent manner [96]. H_2_S production is maximal at low concentrations of Ca^2+^ (~10 nM) [96]. There is no change in the activity of 3MST/CAT pathway in the presence or absence of calmodulin or a calmodulin inhibitor [96]. H_2_S suppresses voltage-gated Ca^2+^ channels in photoreceptor cells by decreasing pH through that activation of vacuolar-type H^+^-ATPase (V-ATPase) in horizontal cells. This in turn leads to the maintenance of intracellular Ca^2+^ in photoreceptor cells at low levels [96]. Excess, strong, long-time light exposure induces retinal degeneration by ROS and the elevation of intracellular concentrations of Ca^2+^ [97,98]. The elevation of intracellular Ca^2+^ results in the inhibition of H_2_S production, thus reducing its protective effects, thereby inducing damage to photoreceptor cells. However, in such conditions, the intraperitoneal administration of an H_2_S donor can suppress photoreceptor cell death [96]. The light-induced increase of TUNEL- and 8-hydroxy-2′-deoxyguanosine positive cells was decreased by the administration of H_2_S [96]. H_2_S protects photoreceptor cells from light-induced retinal degeneration. These reports further indicate a neuroprotective effect of H_2_S, providing a basis for its therapeutic use for neurodegeneration.

## 4. Neuronal Cell Death by NO

### 4.1. Nitric Oxide-Induced Calcium Release (NICR)

NO is a gaseous signaling molecule that is as equally important as Ca^2+^. At least three distinct isoforms of the NOS enzyme are known: including neuronal nitric oxide synthase (nNOS, NOS1), endothelial nitric oxide synthase (eNOS, NOS3), and inducible nitric oxide synthase (iNOS, NOS2) [99]. The first two enzyme isoforms, nNOS and eNOS are activated by elevated intracellular Ca^2+^ [100]. The nNOS isoform is mainly expressed in neurons. The important physiological role of NO that was first identified was its role in the relaxation of blood vessels, following NO release from vascular endothelial cells. NO has been recently shown to be involved in a variety of physiological and pathophysiological functions, including synaptic plasticity and neuronal cell death, in a range of cell types [101,102].

NO activates soluble guanylyl cyclase and subsequent cyclic GMP signaling [103]. NO signaling also regulates other downstream signaling pathways: for instance, it regulates the function of target proteins through the *S*-nitrosylation of cysteine residues [16,104,105]. An increasing number of studies demonstrate the *S*-nitrosylation of proteins by NO, illustrating the important role it plays in a wide range of signaling pathways [18,105].

Ryanodine receptor (RyR) is one of the target proteins that undergoes *S*-nitrosylation [106]. RyRs are large tetrameric channels that control the release of Ca^2+^ from the endoplasmic and sarcoplasmic reticula [107]. Three isoforms (RyR1, RyR2, and RyR3) have been isolated in mammalian tissues. Type 1 RyR (RyR1) is physiologically regulated by voltage-gated Ca^2+^ channels through direct protein–protein interactions in excitation–contraction coupling in skeletal muscle. However, in cardiac muscle, the opening of type 2 RyR (RyR2) is regulated by an influx of Ca^2+^ via voltage-gated Ca^2+^ channels through the Ca^2+^-induced Ca^2+^ release (CICR) mechanism [107]. Further, RyR1 is also expressed in the brain, where we recently identified another mode of intracellular Ca^2+^ mobilization mediated by RyR: the NO-induced Ca^2+^ release (NICR), which is dependent on the *S*-nitrosylation of RyR1 at cysteine 3635 [108]. In Purkinje cells in acute cerebellar slices, NICR is induced by physiological patterns of neuronal activity, and is essential for the induction of cerebellar LTP, which is the reverse process of cerebellar long-term depression (LTD) [108,109]

### 4.2. Involvement of NICR in NO-Induced Neuronal Cell Death

**Figure 3:**
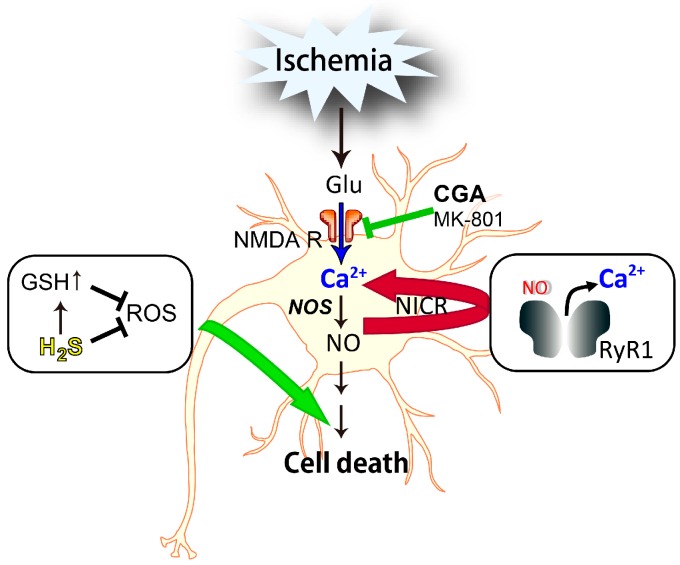
Effects of natural products and gaseous mediators on neuronal cell death. The *N*-methyl-d-aspartate receptor (NMDAR) is linked to downstream molecules, such as nitric oxide synthase (NOS). The activation of NOS leads to the production of nitric oxide (NO), which induces Ca^2+^ release from the endoplasmic reticulum (ER) through *S*-nitrosylation of the type 1 ryanodine receptor (RyR1) and exacerbates neuronal cell death (red arrow). CGA inhibits NMDAR and subsequent influx of Ca^2+^ into the neuronal cytosol (blue arrow), and prevents cell death. Hydrogen sulfide (H_2_S) protects neurons by increasing the levels of glutathione (GSH) and directly suppressing ROS in the mitochondria (green arrow). Abbreviations: Glu, glutamate; NICR, NO-induced Ca^2+^ release; CGA, chlorogenic acid; ROS, reactive oxygen species.

The nNOS-dependent generation of NO is implicated in cerebral ischemia [110]. The NO concentration is estimated to reach micromolar levels in ischemic cerebral tissue, during and after MCAO [111]. Indeed, brain injury after MCAO is significantly milder in mice treated with a nNOS-specific inhibitor and in Nos1^−/−^ mice [112]. Further, infarct volume occurring after ischemia reperfusion was reduced by the administration of dantrolene, a RyR inhibitor [108]. These results suggest that nNOS, as well as RyR, are involved in ischemic brain injury, and NICR, dependent on both nNOS and RyR1, may play a role in ischemic brain injury following reperfusion in the MCAO model [108]. Actually, NICR exacerbates neuronal cell death in the hippocampal CA3 region of kainic acid-induced seizures [113] (Figure 3).

Currently, neuronal cell death by cerebral ischemia is suggested to be induced by the following orders: (1) glutamate release; (2) Ca^2+^ influx-mediated glutamate receptor; (3) NO production by NOS activation; and (4) activation of NO signaling [8,11]. Glutamate-induced neurotoxicity is Ca^2+^-dependent and is mainly mediated through the activation of NMDA receptors [5]. The NO donor induced an increase in intracellular Ca^2+^ in cerebral neurons, and the response was abolished in the presence of dantrolene or in Ryr1^−/−^ mice. There was a significant increase in cerebral neuron death after treatment with an NO donor. In the presence of dantrolene, the NO donor-induced neuronal cell death was significantly attenuated [108]. Therefore, NICR is, at least in part, involved in NO-induced neuronal cell death [108]. Conversely, there was little effect of CGA on NO-induced cell death [68]. It is suggested that the effects of CGA do not affect the downstream pathway of NO leading to the ischemic brain injury (Figure 3). It is also interesting to note that NICR might be involved in mitochondrial dysfunction [113]. The modulation of NO pathways could prevent oxidative damage to neurons, via the inhibition of apoptosis, which is partly induced by NICR.

## 5. Conclusions

Although the etiology of main neurodegenerative diseases is not fully understood yet, glutamate is indicated to be one of the most convincing factors involved in the oxidative stress process that underlies these illnesses. In this paper, we reviewed the neuroprotective and/or neurodegenerative effects of natural products and gaseous mediators. In many case, these molecules affects glutamate-evoked events leading to neuronal cell death. For example, CGA in coffee can inhibit glutamate receptors, thus, playing a neuroprotective role by inhibiting the excessive increase of intracellular Ca^2+^. H_2_S protects neurons from glutamate-induced cell death by recovering the levels of GSH, a major antioxidant, and suppressing the effects of ROS. On the other hand, NO, whose production is initiated by the activation of NMDA receptors by glutamate, induced neurodegenerative effects through NO-induced Ca^2+^ release. Therefore, the developments of drugs mimicking the neuroprotective effects of the natural products or H_2_S, or specifically inhibiting the neurodegenerative effects by NO, may be beneficial for the therapeutic treatment of neurodegenerative diseases.

## Abbreviations

3MST	3-Mercaptopyruvate sulfurtransferase
Aβ	β-amyloid
CA	Caffeic acid
CAPE	Caffeic acid phenethyl ester
CAT	Cysteine aminotransferase
CBS	Cystathionine β-synthase
CFTR	Cystic fibrosis transmembrane conductance regulator
CGA	Chlorogenic acid
CICR	Calcium-induced calcium release
CSE	Cystathionine γ-lyase
DAO	d-amino acid oxidase
DHLA	Dihydrolipoic acid
eNOS	Endothelial nitric oxide synthase
ER	Endoplasmic reticulum
H_2_S	Hydrogen sulfide
iNOS	Inducible nitric oxide synthase
MCAO	Middle cerebral artery occlusion
MeHg	Methylmercury
NICR	Nitric oxide-induced calcium release
NMDA	*N*-methyl d-aspartate
nNOS	Neuronal nitric oxide synthase
NO	Nitric oxide
NOS	Nitric oxide synthase
Nrf2	Nuclear factor erythroid-2 related factor 2
PI	Propidium iodide
ROS	Reactive oxygen species
RyR	Ryanodine receptor

## References

[1] Hardy J., Selkoe D.J. (2002). The amyloid hypothesis of Alzheimer’s disease: Progress and problems on the road to therapeutics. Science.

[2] Prentice H., Modi J.P., Wu J.Y. (2015). Mechanisms of neuronal protection against excitotoxicity, endoplasmic reticulum stress, and mitochondrial dysfunction in stroke and neurodegenerative diseases. Oxid. Med. Cell. Longev..

[3] Thakur S., Sarkar B., Cholia R.P., Gautam N., Dhiman M., Mantha A.K. (2014). APE1/Ref-1 as an emerging therapeutic target for various human diseases: Phytochemical modulation of its functions. Exp. Mol. Med..

[4] Kelsey N.A., Wilkins H.M., Linseman D.A. (2010). Nutraceutical antioxidants as novel neuroprotective agents. Molecules.

[5] Choi D.W., Rothman S.M. (1990). The role of glutamate neurotoxicity in hypoxic-ischemic neuronal death. Annu. Rev. Neurosci..

[6] Manucha W. (2016). Mitochondrial dysfunction associated with nitric oxide pathways in glutamate neurotoxicity. Clin. Investig. Arterioscler..

[7] Szydlowska K., Tymianski M. (2010). Calcium, ischemia and excitotoxicity. Cell Calcium.

[8] Lai T.W., Zhang S., Wang Y.T. (2014). Excitotoxicity and stroke: Identifying novel targets for neuroprotection. Prog. Neurobiol..

[9] Anitha M., Nandhu M.S., Anju T.R., Jes P., Paulose C.S. (2011). Targeting glutamate mediated excitotoxicity in Huntington’s disease: Neural progenitors and partial glutamate antagonist—Memantine. Med. Hypotheses.

[10] Godinez-Rubi M., Rojas-Mayorquin A.E., Ortuno-Sahagun D. (2013). Nitric oxide donors as neuroprotective agents after an ischemic stroke-related inflammatory reaction. Oxid. Med. Cell. Longev..

[11] Kostandy B.B. (2012). The role of glutamate in neuronal ischemic injury: The role of spark in fire. Neurol. Sci..

[12] Nakamura T., Lipton S.A. (2009). Cell death: Protein misfolding and neurodegenerative diseases. Apoptosis.

[13] Stout A.K., Raphael H.M., Kanterewicz B.I., Klann E., Reynolds I.J. (1998). Glutamate-induced neuron death requires mitochondrial calcium uptake. Nat. Neurosci..

[14] Bredt D.S., Snyder S.H. (1994). Nitric oxide: A physiologic messenger molecule. Annu. Rev. Biochem..

[15] Yoneyama M., Kawada K., Shiba T., Ogita K. (2011). Endogenous nitric oxide generation linked to ryanodine receptors activates cyclic GMP/protein kinase G pathway for cell proliferation of neural stem/progenitor cells derived from embryonic hippocampus. J. Pharmacol. Sci..

[16] Hess D.T., Matsumoto A., Kim S.O., Marshall H.E., Stamler J.S. (2005). Protein *S*-nitrosylation: Purview and parameters. Nat. Rev. Mol. Cell Biol..

[17] Nakamura T., Lipton S.A. (2016). Protein *S*-nitrosylation as a therapeutic target for neurodegenerative diseases. Trends Pharmacol. Sci..

[18] Nakamura T., Tu S., Akhtar M.W., Sunico C.R., Okamoto S., Lipton S.A. (2013). Aberrant protein *S*-nitrosylation in neurodegenerative diseases. Neuron.

[19] Asard H., Banerjee R. (2007). Ascorbate. Redox Biochemistry.

[20] Banhegyi G., Braun L., Csala M., Puskas F., Mandl J. (1997). Ascorbate metabolism and its regulation in animals. Free Radic. Biol. Med..

[21] Della Penna D., Pogson B.J. (2006). Vitamin synthesis in plants: Tocopherols and carotenoids. Annu. Rev. Plant Biol..

[22] Li A.N., Li S., Zhang Y.J., Xu X.R., Chen Y.M., Li H.B. (2014). Resources and biological activities of natural polyphenols. Nutrients.

[23] Brglez Mojzer E., Knez Hrncic M., Skerget M., Knez Z., Bren U. (2016). Polyphenols: Extraction Methods, Antioxidative Action, Bioavailability and Anticarcinogenic Effects. Molecules.

[24] Barycki J.J., Banerjee R. (2007). Glutathione. Redox Biochemicstry.

[25] Circu M.L., Aw T.Y. (2012). Glutathione and modulation of cell apoptosis. Biochim. Biophys. Acta.

[26] Jones D.P., Carlson J.L., Mody V.C., Cai J., Lynn M.J., Sternberg P. (2000). Redox state of glutathione in human plasma. Free Radic. Biol. Med..

[27] Meister A., Anderson M.E. (1983). Glutathione. Annu. Rev. Biochem..

[28] Morikawa T., Yasuno R., Wada H. (2001). Do mammalian cells synthesize lipoic acid? Identification of a mouse cDNA encoding a lipoic acid synthase located in mitochondria. FEBS Lett..

[29] Reed L.J., Leach F.R., Koike M. (1958). Studies on a lipoic acid-activating system. J. Biol. Chem..

[30] Smith A.R., Shenvi S.V., Widlansky M., Suh J.H., Hagen T.M. (2004). Lipoic acid as a potential therapy for chronic diseases associated with oxidative stress. Curr. Med. Chem..

[31] Abe K., Kimura H. (1996). The possible role of hydrogen sulfide as an endogenous neuromodulator. J. Neurosci..

[32] Higdon J.V., Frei B. (2006). Coffee and health: A review of recent human research. Crit. Rev. Food Sci. Nutr..

[33] Andersen L.F., Jacobs D.R.J., Carlsen M.H., Blomhoff R. (2006). Consumption of coffee is associated with reduced risk of death attributed to inflammatory and cardiovascular diseases in the Iowa Women’s Health Study. Am. J. Clin. Nutr..

[34] Freedman N.D., Park Y., Abnet C.C., Hollenbeck A.R., Sinha R. (2012). Association of coffee drinking with total and cause-specific mortality. N. Engl. J. Med..

[35] O’Keefe J.H., Bhatti S.K., Patil H.R., DiNicolantonio J.J., Lucan S.C., Lavie C.J. (2013). Effects of habitual coffee consumption on cardiometabolic disease, cardiovascular health, and all-cause mortality. J. Am. Coll. Cardiol..

[36] Rebello S.A., van Dam R.M. (2013). Coffee consumption and cardiovascular health: Getting to the heart of the matter. Curr. Cardiol. Rep..

[37] Kokubo Y., Iso H., Saito I., Yamagishi K., Yatsuya H., Ishihara J., Inoue M., Tsugane S. (2013). The impact of green tea and coffee consumption on the reduced risk of stroke incidence in Japanese population: The Japan public health center-based study cohort. Stroke.

[38] Larsson S.C., Virtamo J., Wolk A. (2011). Coffee consumption and risk of stroke in women. Stroke.

[39] Lopez-Garcia E., Rodriguez-Artalejo F., Rexrode K.M., Logroscino G., Hu F.B., van Dam R.M. (2009). Coffee consumption and risk of stroke in women. Circulation.

[40] Croft K.D. (1998). The chemistry and biological effects of flavonoids and phenolic acids. Ann. N. Y. Acad. Sci..

[41] Sul D., Kim H.S., Lee D., Joo S.S., Hwang K.W., Park S.Y. (2009). Protective effect of caffeic acid against β-amyloid-induced neurotoxicity by the inhibition of calcium influx and tau phosphorylation. Life Sci..

[42] Lane R.M., Potkin S.G., Enz A. (2006). Targeting acetylcholinesterase and butyrylcholinesterase in dementia. Int. J. Neuropsychopharmacol..

[43] Oboh G., Agunloye O.M., Akinyemi A.J., Ademiluyi A.O., Adefegha S.A. (2013). Comparative study on the inhibitory effect of caffeic and chlorogenic acids on key enzymes linked to Alzheimer’s disease and some pro-oxidant induced oxidative stress in rats’ brain-in vitro. Neurochem. Res..

[44] Huang Y., Jin M., Pi R., Zhang J., Chen M., Ouyang Y., Liu A., Chao X., Liu P., Liu J. (2013). Protective effects of caffeic acid and caffeic acid phenethyl ester against acrolein-induced neurotoxicity in HT22 mouse hippocampal cells. Neurosci. Lett..

[45] Scapagnini G., Vasto S., Abraham N.G., Caruso C., Zella D., Fabio G. (2011). Modulation of Nrf2/ARE pathway by food polyphenols: A nutritional neuroprotective strategy for cognitive and neurodegenerative disorders. Mol. Neurobiol..

[46] Wei X., Ma Z., Fontanilla C.V., Zhao L., Xu Z.C., Taggliabraci V., Johnstone B.H., Dodel R.C., Farlow M.R., Du Y. (2008). Caffeic acid phenethyl ester prevents cerebellar granule neurons (CGNs) against glutamate-induced neurotoxicity. Neuroscience.

[47] Dos Santos N.A., Martins N.M., Silva Rde B., Ferreira R.S., Sisti F.M., dos Santos A.C. (2014). Caffeic acid phenethyl ester (CAPE) protects PC12 cells from MPP^+^ toxicity by inducing the expression of neuron-typical proteins. Neurotoxicology.

[48] Kim J.H., Wang Q., Choi J.M., Lee S., Cho E.J. (2015). Protective role of caffeic acid in an Aβ_25–35_-induced-induced Alzheimer’s disease model. Nutr. Res. Pract..

[49] Barros Silva R., Santos N.A., Martins N.M., Ferreira D.A., Barbosa F., Oliveira Souza V.C., Kinoshita A., Baffa O., del-Bel E., Santos A.C. (2013). Caffeic acid phenethyl ester protects against the dopaminergic neuronal loss induced by 6-hydroxydopamine in rats. Neuroscience.

[50] Liang G., Shi B., Luo W., Yang J. (2015). The protective effect of caffeic acid on global cerebral ischemia-reperfusion injury in rats. Behav. Brain Funct..

[51] Fredholm B.B., Bättig K., Holmén J., Nehlig A., Zvartau E.E. (1999). Actions of caffeine in the brain with special reference to factors that contribute to its widespread use. Pharmacol. Rev..

[52] Dunwiddie T.V., Masino S.A. (2001). The role and regulation of adenosine in the central nervous system. Annu. Rev. Neurosci..

[53] Ascherio A., Zhang S.M., Hernan M.A., Kawachi I., Colditz G.A., Speizer F.E., Willett W.C. (2001). Prospective study of caffeine consumption and risk of Parkinson’s disease in men and women. Ann. Neurol..

[54] Benedetti M.D., Bower J.H., Maraganore D.M., McDonnell S.K., Peterson B.J., Ahlskog J.E., Schaid D.J., Rocca W.A. (2000). Smoking, alcohol, and coffee consumption preceding Parkinson’s disease: A case-control study. Neurology.

[55] Clifford M.N., Knight S., Surucu B., Kuhnert N. (2006). Characterization by LC-MS^n^ of four new classes of chlorogenic acids in green coffee beans: Dimethoxycinnamoylquinic acids, diferuloylquinic acids, caffeoyl-dimethoxycinnamoylquinic acids, and feruloyl-dimethoxycinnamoylquinic acids. J. Agric. Food Chem..

[56] Farah A., Monteiro M.C., Calado V., Franca A.S., Trugo L.C. (2006). Correlation between cup quality and chemical attributes of Brazilian coffee. Food Chem..

[57] Upadhyay R., Mohan Rao L.J. (2013). An outlook on chlorogenic acids-occurrence, chemistry, technology, and biological activities. Crit. Rev. Food Sci. Nutr..

[58] Feng R., Lu Y., Bowman L.L., Qian Y., Castranova V., Ding M. (2005). Inhibition of activator protein-1, NF-κB, and MAPKs and induction of phase 2 detoxifying enzyme activity by chlorogenic acid. J. Biol. Chem..

[59] Li Y., Shi W., Li Y., Zhou Y., Hu X., Song C., Ma H., Wang C., Li Y. (2008). Neuroprotective effects of chlorogenic acid against apoptosis of PC12 cells induced by methylmercury. Environ. Toxicol. Pharmacol..

[60] Weng C.J., Yen G.C. (2012). Chemopreventive effects of dietary phytochemicals against cancer invasion and metastasis: Phenolic acids, monophenol, polyphenol, and their derivatives. Cancer Treat. Rev..

[61] Zang L.Y., Cosma G., Gardner H., Castranova V., Vallyathan V. (2003). Effect of chlorogenic acid on hydroxyl radical. Mol. Cell. Biochem..

[62] Lee K., Lee J.S., Jang H.J., Kim S.M., Chang M.S., Park S.H., Kim K.S., Bae J., Park J.W., Lee B. (2012). Chlorogenic acid ameliorates brain damage and edema by inhibiting matrix metalloproteinase-2 and 9 in a rat model of focal cerebral ischemia. Eur. J. Pharmacol..

[63] Cho E.S., Jang Y.J., Hwang M.K., Kang N.J., Lee K.W., Lee H.J. (2009). Attenuation of oxidative neuronal cell death by coffee phenolic phytochemicals. Mutat. Res..

[64] Pavlica S., Gebhardt R. (2005). Protective effects of ellagic and chlorogenic acids against oxidative stress in PC12 cells. Free Radic. Res..

[65] Ali S.F., LeBel C.P., Bondy S.C. (1992). Reactive oxygen species formation as a biomarker of methylmercury and trimethyltin neurotoxicity. Neurotoxicology.

[66] Sarafian T.A., Vartavarian L., Kane D.J., Bredesen D.E., Verity M.A. (1994). *Bcl-2* expression decreases methyl mercury-induced free-radical generation and cell killing in a neural cell line. Toxicol. Lett..

[67] Ito H., Sun X.L., Watanabe M., Okamoto M., Hatano T. (2008). Chlorogenic acid and its metabolite m-coumaric acid evoke neurite outgrowth in hippocampal neuronal cells. Biosci. Biotechnol. Biochem..

[68] Mikami Y., Yamazawa T. (2015). Chlorogenic acid, a polyphenol in coffee, protects neurons against glutamate neurotoxicity. Life Sci..

[69] Kanemaru K., Kubota J., Sekiya H., Hirose K., Okubo Y., Iino M. (2013). Calcium-dependent *N*-cadherin up-regulation mediates reactive astrogliosis and neuroprotection after brain injury. Proc. Natl. Acad. Sci. USA.

[70] Nakamura N., Yamazawa T., Okubo Y., Iino M. (2009). Temporal switching and cell-to-cell variability in Ca^2+^ release activity in mammalian cells. Mol. Syst. Biol..

[71] Goodwin L.R., Francom D., Dieken F.P., Taylor J.D., Warenycia M.W., Reiffenstein R.J., Dowling G. (1989). Determination of sulfide in brain tissue by gas dialysis/ion chromatography: Postmortem studies and two case reports. J. Anal. Toxicol..

[72] Savage J.C., Gould D.H. (1990). Determination of sulfide in brain tissue and rumen fluid by ion-interaction reversed-phase high-performance liquid chromatography. J. Chromatogr..

[73] Warenycia M.W., Goodwin L.R., Benishin C.G., Reiffenstein R.J., Francom D.M., Taylor J.D., Dieken F.P. (1989). Acute hydrogen sulfide poisoning. Demonstration of selective uptake of sulfide by the brainstem by measurement of brain sulfide levels. Biochem. Pharmacol..

[74] Hosoki R., Matsuki N., Kimura H. (1997). The possible role of hydrogen sulfide as an endogenous smooth muscle relaxant in synergy with nitric oxide. Biochem. Biophys. Res. Commun..

[75] Shibuya N., Tanaka M., Yoshida M., Ogasawara Y., Togawa T., Ishii K., Kimura H. (2009). 3-Mercaptopyruvate sulfurtransferase produces hydrogen sulfide and bound sulfane sulfur in the brain. Antioxid. Redox Signal..

[76] Mikami Y., Shibuya N., Kimura Y., Nagahara N., Ogasawara Y., Kimura H. (2011). Thioredoxin and dihydrolipoic acid are required for 3-mercaptopyruvate sulfurtransferase to produce hydrogen sulfide. Biochem. J..

[77] Shibuya N., Koike S., Tanaka M., Ishigami-Yuasa M., Kimura Y., Ogasawara Y., Fukui K., Nagahara N., Kimura H. (2013). A novel pathway for the production of hydrogen sulfide from d-cysteine in mammalian cells. Nat. Commun..

[78] Kimura Y., Mikami Y., Osumi K., Tsugane M., Oka J., Kimura H. (2013). Polysulfides are possible H_2_S-derived signaling molecules in rat brain. FASEB J..

[79] Kimura Y., Toyofuku Y., Koike S., Shibuya N., Nagahara N., Lefer D., Ogasawara Y., Kimura H. (2015). Identification of H_2_S_3_ and H_2_S produced by 3-mercaptopyruvate sulfurtransferase in the brain. Sci. Rep..

[80] Nagai Y., Tsugane M., Oka J., Kimura H. (2004). Hydrogen sulfide induces calcium waves in astrocytes. FASEB J..

[81] Shibuya N., Mikami Y., Kimura Y., Nagahara N., Kimura H. (2009). Vascular endothelium expresses 3-mercaptopyruvate sulfurtransferase and produces hydrogen sulfide. J. Biochem..

[82] Yang G., Wu L., Jiang B., Yang W., Qi J., Cao K., Meng Q., Mustafa A.K., Mu W., Zhang S. (2008). H_2_S as a physiologic vasorelaxant: Hypertension in mice with deletion of cystathionine γ-lyase. Science.

[83] Zhao W., Zhang J., Lu Y., Wang R. (2001). The vasorelaxant effect of H*_2_*S as a novel endogenous gaseous K_ATP_ channel opener. EMBO J..

[84] Ishii I., Akahoshi N., Yamada H., Nakano S., Izumi T., Suematsu M. (2010). Cystathionine γ-lyase-deficient mice require dietary cysteine to protect against acute lethal myopathy and oxidative injury. J. Biol. Chem..

[85] Kimura Y., Kimura H. (2004). Hydrogen sulfide protects neurons from oxidative stress. FASEB J..

[86] Kimura Y., Goto Y., Kimura H. (2010). Hydrogen sulfide increases glutathione production and suppresses oxidative stress in mitochondria. Antioxid. Redox Signal..

[87] Whiteman M., Armstrong J.S., Chu S.H., Jia-Ling S., Wong B.S., Cheung N.S., Halliwell B., Moore P.K. (2004). The novel neuromodulator hydrogen sulfide: An endogenous peroxynitrite “scavenger”?. J. Neurochem..

[88] Whiteman M., Cheung N.S., Zhu Y.Z., Chu S.H., Siau J.L., Wong B.S., Armstrong J.S., Moore P.K. (2005). Hydrogen sulphide: A novel inhibitor of hypochlorous acid-mediated oxidative damage in the brain?. Biochem. Biophys. Res. Commun..

[89] Elrod J.W., Calvert J.W., Morrison J., Doeller J.E., Kraus D.W., Tao L., Jiao X., Scalia R., Kiss L., Szabo C. (2007). Hydrogen sulfide attenuates myocardial ischemia-reperfusion injury by preservation of mitochondrial function. Proc. Natl. Acad. Sci. USA.

[90] Calvert J.W., Jha S., Gundewar S., Elrod J.W., Ramachandran A., Pattillo C.B., Kevil C.G., Lefer D.J. (2009). Hydrogen sulfide mediates cardioprotection through Nrf2 signaling. Circ. Res..

[91] Calvert J.W., Coetzee W.A., Lefer D.J. (2010). Novel insights into hydrogen sulfide-mediated cytoprotection. Antioxid. Redox Signal..

[92] Suh J.H., Shenvi S.V., Dixon B.M., Liu H., Jaiswal A.K., Liu R.M., Hagen T.M. (2004). Decline in transcriptional activity of Nrf2 causes age-related loss of glutathione synthesis, which is reversible with lipoic acid. Proc. Natl. Acad. Sci. USA.

[93] Koike S., Ogasawara Y., Shibuya N., Kimura H., Ishii K. (2013). Polysulfide exerts a protective effect against cytotoxicity caused by *t*-buthylhydroperoxide through Nrf2 signaling in neuroblastoma cells. FEBS Lett..

[94] Kimura Y., Dargusch R., Schubert D., Kimura H. (2006). Hydrogen sulfide protects HT22 neuronal cells from oxidative stress. Antioxid. Redox Signal..

[95] Johansen D., Ytrehus K., Baxter G.F. (2006). Exogenous hydrogen sulfide (H_2_S) protects against regional myocardial ischemia-reperfusion injury-Evidence for a role of K ATP channels. Basic Res. Cardiol..

[96] Mikami Y., Shibuya N., Kimura Y., Nagahara N., Yamada M., Kimura H. (2011). Hydrogen sulfide protects the retina from light-induced degeneration by the modulation of Ca^2+^ influx. J. Biol. Chem..

[97] Noell W.K., Walker V.S., Kang B.S., Berman S. (1966). Retinal damage by light in rats. Investig. Ophthalmol..

[98] Wenzel A., Grimm C., Samardzija M., Reme C.E. (2005). Molecular mechanisms of light-induced photoreceptor apoptosis and neuroprotection for retinal degeneration. Prog. Retin. Eye Res..

[99] Hollenberg S.M., Cinel I. (2009). Bench-to-bedside review: Nitric oxide in critical illness-update 2008. Crit. Care.

[100] Alderton W.K., Cooper C.E., Knowles R.G. (2001). Nitric oxide synthases: Structure, function and inhibition. Biochem. J..

[101] Ricciardolo F.L., Sterk P.J., Gaston B., Folkerts G. (2004). Nitric oxide in health and disease of the respiratory system. Physiol. Rev..

[102] Pacher P., Beckman J.S., Liaudet L. (2007). Nitric oxide and peroxynitrite in health and disease. Physiol. Rev..

[103] Friebe A., Koesling D. (2003). Regulation of nitric oxide-sensitive guanylyl cyclase. Circ. Res..

[104] Shahani N., Sawa A. (2011). Nitric oxide signaling and nitrosative stress in neurons: Role for *S*-nitrosylation. Antioxid. Redox Signal..

[105] Jaffrey S.R., Erdjument-Bromage H., Ferris C.D., Tempst P., Snyder S.H. (2001). Protein *S*-nitrosylation: A physiological signal for neuronal nitric oxide. Nat. Cell Biol..

[106] Eu J.P., Sun J., Xu L., Stamler J.S., Meissner G. (2000). The skeletal muscle calcium release channel: Coupled O_2_ sensor and NO signaling functions. Cell.

[107] Endo M. (2009). Calcium-induced calcium release in skeletal muscle. Physiol. Rev..

[108] Kakizawa S., Yamazawa T., Chen Y., Ito A., Murayama T., Oyamada H., Kurebayashi N., Sato O., Watanabe M., Mori N. (2012). Nitric oxide-induced calcium release via ryanodine receptors regulates neuronal function. EMBO J..

[109] Kakizawa S., Yamazawa T., Iino M. (2013). Nitric oxide-induced calcium release: Activation of type 1 ryanodine receptor by endogenous nitric oxide. Channels (Austin).

[110] Iadecola C. (1997). Bright and dark sides of nitric oxide in ischemic brain injury. Trends Neurosci..

[111] Malinski T., Bailey F., Zhang Z.G., Chopp M. (1993). Nitric oxide measured by a porphyrinic microsensor in rat brain after transient middle cerebral artery occlusion. J. Cereb. Blood Flow Metab..

[112] Huang Z., Huang P.L., Panahian N., Dalkara T., Fishman M.C., Moskowitz M.A. (1994). Effects of cerebral ischemia in mice deficient in neuronal nitric oxide synthase. Science.

[113] Mikami Y., Kanemaru K., Okubo Y., Nakaune T., Suzuki J., Shibata K., Sugiyama H., Koyama R., Murayama T., Ito A. (2016). Nitric oxide-induced activation of the type 1 ryanodine receptor receptor is critical for epileptic seizure-induced neuronal cell death. EBioMedicine.

